# Cloning, functional expression, and pharmacological characterization of inwardly rectifying potassium channels (Kir) from *Apis mellifera*

**DOI:** 10.1038/s41598-024-58234-0

**Published:** 2024-04-03

**Authors:** Fabien Sourisseau, Chaimaa Chahine, Valérie Pouliot, Thierry Cens, Pierre Charnet, Mohamed Chahine

**Affiliations:** 1 CERVO Brain Research Centre, 2601, chemin de la Canardière, Quebec City, QC G1J 2G3 Canada; 2 Institut des Biomolécules Max Mousseron (IBMM), CNRS UMR 5247, 1919 Route de Mende, Montpellier, France; 3 Department of Medicine, Faculty of Medicine, Université Laval, Quebec City, QC Canada

**Keywords:** Potassium channels, Kir channels, Expression, Cloning, Insects, *Apis mellifera*, Insecticides, VU041, Ion channels in the nervous system, Biophysics, Molecular biology, Neuroscience

## Abstract

Potassium channels belong to the super family of ion channels and play a fundamental role in cell excitability. Kir channels are potassium channels with an inwardly rectifying property. They play a role in setting the resting membrane potential of many excitable cells including neurons. Although putative Kir channel family genes can be found in the *Apis mellifera* genome, their functional expression, biophysical properties, and sensitivity to small molecules with insecticidal activity remain to be investigated. We cloned six Kir channel isoforms from *Apis mellifera* that derive from two Kir genes, AmKir1 and AmKir2, which are present in the *Apis mellifera* genome. We studied the tissue distribution, the electrophysiological and pharmacological characteristics of three isoforms that expressed functional currents (AmKir1.1, AmKir2.2, and AmKir2.3). AmKir1.1, AmKir2.2, and AmKir2.3 isoforms exhibited distinct characteristics when expressed in *Xenopus* oocytes. AmKir1.1 exhibited the largest potassium currents and was impermeable to cesium whereas AmKir2.2 and AmKir2.3 exhibited smaller currents but allowed cesium to permeate. AmKir1 exhibited faster opening kinetics than AmKir2. Pharmacological experiments revealed that both AmKir1.1 and AmKir2.2 are blocked by the divalent ion barium, with IC_50_ values of 10^−5^ and 10^−6^ M, respectively. The concentrations of VU041, a small molecule with insecticidal properties required to achieve a 50% current blockade for all three channels were higher than those needed to block Kir channels in other arthropods, such as the aphid *Aphis gossypii* and the mosquito *Aedes aegypti*. From this, we conclude that *Apis mellifera* AmKir channels exhibit lower sensitivity to VU041.

## Introduction

Nerve impulse propagation takes place through trains of action potentials (AP) triggered when a specific membrane potential threshold is reached^1^. Potassium channels play a crucial role in this impulse activity of various excitable cells, including neurons. These channels are essential for establishing the resting membrane potential and for the generation, propagation, and regulation of AP firing. Kir channels belong to the large family of potassium channels but lack the voltage sensor domain. As a result, Kir channels do not exhibit voltage-dependent activity or activation thresholds. They were named Kir due to their unique inwardly rectifying property, which restricts potassium permeability at more positive voltages. This inwardly rectifying pattern has been attributed to the blockade of the intracellular pore of these channels by various factors such as endogenous polyamines like spermine and spermidine and intracellular cations like magnesium^2,3^. These factors obstruct the channel pore from the intracellular side when the membrane potential is more positive, leading to a decrease in the outward current^4^. Pharmacologically, Kir channels show sensitivity to tetra-ethyl-ammonium (TEA) and 4-aminopyridine (4-AP) and, more specifically, to certain cations such as barium, which is known to be a universal Kir channel blocker^5^. Kir channels play an important role in the physiology of arthropods. They have been recently implicated in the functioning of salivary glands and the structure of Malpighian tubules in the mosquito *Aedes aegypti*, making them potential targets for the development of new insecticides that disrupt potassium homeostasis in the hemolymph, leading to the degeneration of Malpighian tubules in female mosquitoes^6^. Similar effects have been observed in the aphid Aphis gossypii^7^. *Drosophila melanogaster* is another extensively studied arthropod and, like mosquitoes, appears to express three Kir channels in its salivary glands^8,9^. Within insects, Kir channels are attributed to various physiological roles. Kir1 and Kir2 are generally associated within the physiological function of Malpighian tubules^10^, salivary glands^9^, immune response or nervous system^11^. Although Kir3 is highly expressed in Malpighian tubules, specifically targeting Kir3 or Kir1 in the *Drosophila* wings disrupts their morphogenesis^10,12^.

Surprisingly, little is known about the biophysical and pharmacological properties of these channels in the honeybee *Apis mellifera*. Considering the alarming ecological consequences for arthropods and the impact of certain insecticides on their Kir channels, it is of great interest to characterize the expression of these channels in *Apis mellifera* and examine how they are affected by these insecticides. Previous studies have shown that insecticides have adverse effects on some Kir channels subtypes such as K_ATP_ of *Apis mellifera*, resulting in damage to the dorsal vessel (equivalent to the aortic circulatory system in mammals), including the heart, and potential viral protection of honeybees^13–15^. Additionally, VU041 (C_19_H_20_F_3_N_3_O), a quinoline derivative, that was developed to control mosquitoes, has been reported to be non-lethal to honeybees^16^. However, our understanding of *Apis mellifera* Kir (AmKir) channels, whether in terms of structure, electrophysiology, or pharmacology, is limited. Given the fundamental importance of these channels in the physiology of arthropods, it is essential to characterize the biophysical properties of the AmKir channels and investigate the effect of small molecule with insecticidal properties on AmKir currents by pharmacological analyses. This article contributes to a study aimed at understanding the electrophysiology of Kir channels in the honeybee, which we have cloned and expressed. Additionally, we present our observations regarding the effects of the small molecule VU041 on these channels.

## Methods

### Cloning, plasmid constructs, and RNA synthesis

Full length sequences for cloning were obtained using NCBI Gene-Base and Hymenoptera Genome Database. The full-length sequence of both AmKir1 and AmKir2 channels were synthesized by Bio Basic Inc. and cloned into the pPOL_NotI plasmid. Plasmids were linearized with NotI. Messenger RNAs were then synthesized and joined to a Cap protein using HiScribe® T7 Quick High Yield RNA Synthesis kits (New England Biolabs, Vaccina Capping System). *Apis mellifera* presents two types of AmKir channels: AmKir1 (Accession gene ID 408462, composed of AmKir1.1 and AmKir1.2 isoforms) and AmKir2 (Accession gene ID 408463, composed of AmKir2.1, AmKir2.2, AmKir2.3, and AmKir2.4 isoforms).

### RT-PCR

Total RNA was extracted from pooled tissues originating from a honeybee colony (adult legs, heads, brains, ganglia, viscera, muscles, antennae, and larvae) using the Trizol reagent protocol (Life Technologies, Trizol). The total RNA from different tissues was treated with RNase-free DNAseI (New England Biolabs). Subsequently, cDNA was synthesized using ProtoScript® II First Strand cDNA Synthesis kits (New England Biolabs). PCR amplification with oligonucleotide primers was then carried out to obtain cDNA preparations corresponding to the AmKir isotypes. The resulting cDNA preparations were run on agarose gels to assess the expression of the genes of interest in the investigated tissues. Control experiments were conducted by replacing cDNA samples with water. Primer sequences are listed Table 1. No specific oligonucleotide for AmKir2.2 could be developed to assess quantitative expression of this isoform due to the high sequence similarity between AmKir2.1 and AmKir2.2 (Fig. S1).

**Table 1:** Oligonucleotide list used for PCR experiments.

Gene	Primers (5′–3′)		Tm (°C)	Length (pb)
	Forward	Reverse		
*AmKir1.1*	TCAGAGACAGCAACAGAGCAG	GTTGGTCAGGATTCCCCGTT	54	149
*AmKir1.2*	GGAAAGATCCACTTGGGCGA	CTCGTCTGCCGGTATTTTTGC	54	140
*AmKir2.1*	AGCTGGAAAACGCGGGAAAA	CACCATTCCTCGTCCCCGAA	58	124
*AmKir2.3*	GGAAACGTTGCGAAAGATCCA	GGTACCGACTCCTGCTTCT	54	151
*AmKir2.4*	TGCGGAGAAATCCATCGAGTC	GGTCGTGAAGATGTCTCGCA	54	146
*GAPDH*	CACCTTCTGCAAAATTATGGCG	ACCTTTGCCAAGTCTAACTGTTAA	58	188
*RPS18*	GATTCCCGATTGGTTTTTGAATAG	AACCCCAATAATGACGCAAACC	58	152

*Tm* melting temperature.

### Collection, injection, and culture of *Xenopus* oocytes

Female *Xenopus laevis* frogs were anesthetized with 1.5 mg/mL of tricaine (E10521, Millipore Sigma), a topical anesthetic. Two or three ovarian lobes were surgically removed. The follicular cells surrounding the oocytes were removed by incubation at 20 °C for 1 h in 0-calcium oocyte medium (96 mM NaCl, 2 mM KCl, 1 mM MgCl_2_, 5 mM HEPES, pH 7.6) containing 1 mg/mL of type 1A collagenase (Millipore Sigma), as previously described^17^. The medium was then replaced by fresh medium, and the incubation was continued for a further 1 h. Stage IV round, large oocytes were washed with 0-calcium oocyte medium and then with ½-diluted Leibovitz’s L-15 medium (11415064, Gibco, ThermoFisher Scientific) supplemented with 15 mM HEPES, 1 mM glutamine, and 60 μg/mL of gentamicin (pH 7.6). The oocytes were stored at 18 °C in this medium until used. Selected oocytes were then microinjected with 25 ng of RNA. The oocytes were used for experiments 1 to 3-days post-injection.

### HEK 293 Cell culture and AmKir1.1 plasmid cloning

Human Embryonic kidney 293 (HEK293T) cells were also used to express AmKir1.1 channel. The cells were grown in high glucose Dulbecco’s modified Eagle’s medium supplemented with 10% fetal bovine serum and 1% streptomycin at 37 °C—5% CO_2_ atmosphere as previously reported^18^. The *Apis Mellifera* AmKir1.1 channel has been assembled in a pIRES_EGFP plasmid (with the Kit NEBuilder HiFi DNA Assemby Master mix: NEB#E2621S) from the amplified sequence of AmKir1.1 channel in our pPOL_NotI_AmKir1.1 plasmid between EcoRI and BamHI site (See Method—Cloning, plasmid constructs, and RNA synthesis). Cells were transfected with 1 µg of plasmid pIRES-EGFP_AmKir1.1 in 10-cm cell culture dishes using the transfection with calcium phosphate method reported previously^19^.

### Current recordings and pharmacology

Current recordings of oocytes injected with AmKir1.1 were processed one day after injection, while those injected with AmKir2.2 and AmKir2.3 were processed two and three days later, respectively, due to a delay in their expression. The oocytes were observed using an SMZ645 Stereo Microscope (Nikon). The current recordings were processed using an Oocyte Clamp Amplifier (OC-725C, Warner Instruments) and an Axon™ Digidata 1550B Low-Noise Data Acquisition System (Molecular Devices). The recordings were amplified 10 times and were filtered at 0.1 kHz using an LPF-100B low pass filter (Warner Instruments). They were then recorded using pClamp 11.2 software (Molecular Devices). The reference electrodes were managed using an Oocyte Bath Clamp GND 7251 I system (Warner Instruments). Current recordings were performed when the currents were stable and without leak subtraction. The cells were polarized at a holding potential of − 80 mV and were then subjected to pulses ranging from − 150 to + 50 mV for 500 ms, followed by a return to the − 80 mV holding potential. The sweeps were repeated every 2 s in 10-mV increments for each new sweep. Half-time to peak data have been obtained by dividing by half the total time needed to get the maximum current observed on Clampfit 10.7 on I-V curves in condition Ringer 96 mM KCl. Buffer composition used to modulate potassium driving force and ionic permeability within *Xenopus* oocytes are listed in Tables 2 and 3, respectively. Buffer composition used in HEK293T experiment are listed in Table 4. The pharmacology experiments with barium and VU041 were performed with 96 mM KCl Ringer’s solution and increasing concentrations of the two pharmacological agents ranging from 10^−7^ to 10^−2^ M for barium (Sigma) and from 10^−8^ to 10^−4^ M for VU041. VU041 was obtained from Dr. Swale (Department of Entomology, Louisiana State University Agricultural Center, Baton Rouge, LA, USA) and from Millipore Sigma.

**Table 2:** Chemical composition (in mM) of solution used in *Xenopus* oocyte for potassium driving force experiment.

(mM)	KCl	NaCl	CaCl_2_	MgCl_2_	HEPES
Ringer 2 mM KCl	2	116	1.8	2	5
Ringer 25 mM KCl	25	96	1.8	2	5
Ringer 96 mM KCl	96	25	1.8	2	5

The pH of all solutions was adjusted to 7.6 ± 0.05 with NaOH (Ringer 2 mM KCl and Ringer 25 mM KCl) and KOH (Ringer 96 mM KCl).

**Table 3:** Chemical composition (in mM) of solution used in *Xenopus* oocyte for ionic permeability experiment.

(mM)	KCl	NaCl	CaCl_2_	MgCl_2_	HEPES	NMDG	TlCl	RbCl	CsCl	LiCl
Tl 10 mM	0	0	1.8	2	5	108	10	0	0	0
K 10 mM	10	0	1.8	2	5	108	0	0	0	0
Rb 10 mM	0	0	1.8	2	5	108	0	10	0	0
Na 10 mM	0	10	1.8	2	5	108	0	0	0	0
Cs 10 mM	0	0	1.8	2	5	108	0	0	10	0
Li 10 mM	0	0	1.8	2	5	108	0	0	0	10

The pH of all solutions was adjusted to 7.6 ± 0.05 with methanesulfonic acid. NMDG = *N*-Methyl-d-glucamine. For 96 mM of cation experiments, NMDG concentration were 22 mM and tested ion concentration were 96 mM.

**Table 4:** Chemical composition (in mM) of solution used in HEK293T for AmKir1.1 pharmacological interaction with VU041.

	KCl	NaCl	CaCl_2_	MgCl_2_	HEPES	EGTA	Glucose
Extracellular	25	115	2	1	10	0	5
Intracellular	135	0	0	2	10	1	0

The pH of all solutions was adjusted to 7.4 ± 0.05 with NaOH (Extracellular) or KOH (Intracellular); *EGTA* Ethylene-bis(oxyethylenenitrilo)tetraacetic acid.

### Statistical analysis

All statistical analyses were performed using PRISM10 software (GraphPad, Ca, USA). The normality distribution was determined by using the D’Agostino-Person normality test. Data are expressed in Mean + Standard Error of the Mean (SEM). Electrophysiological data replicates are represented as an “n” that represents the number of oocytes recorded. Two independent variables were analyzed with a two-way ANOVA with a Tukey’s multiple comparisons test was used. When more than two means are compared at the same time, a one-way ANOVA with a Tukey’s multiple comparisons test was used. Otherwise, a two-tailed unpaired Student’s t-test was used to compare one mean to another. All the statistical tests were performed while aiming for a 95% confidence interval, and the differences were considered significant when the resulting p value was considered under 0.05.

## Results

### Relative tissue expression and AmKir sequences in *Apis mellifera*

**Figure 1:**
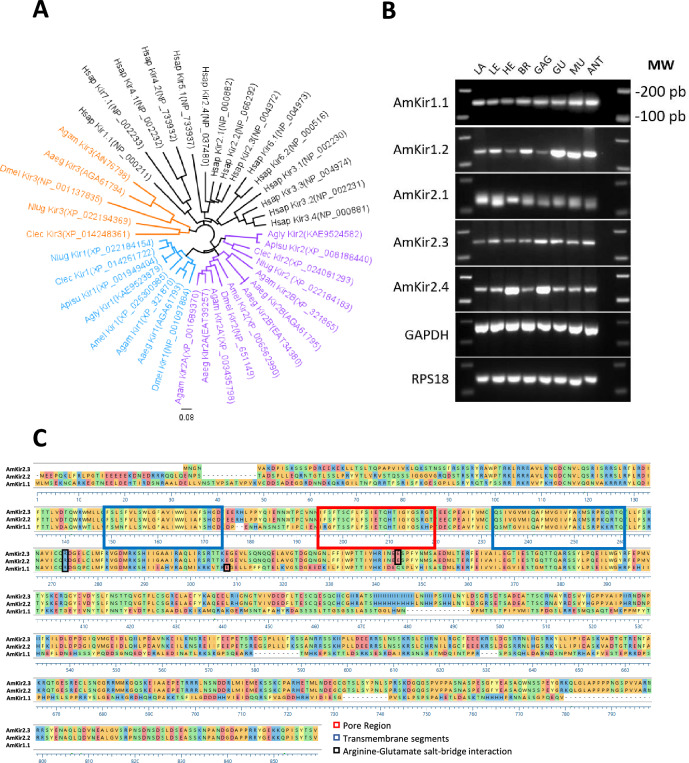
(**A**) Neighbor-joining phylogenic tree of amino acid sequences encoding Kir channel subunits in insects and humans. Geneious Prime 2023.2.2 (www.geneious.com) was used to construct the tree. The Genbank® accession numbers of the Kir sequences are shown in parentheses. Underlines denote *Apis mellifera* Kir channel subunits. The abbreviations of the species are as follows: Aag: *Aedes aegypti*, Agam: *Anopheles gambiae*, Agly: *Aphis glycines*, Amel: *Apis mellifera*, Apisu: *Acyrthosiphon pisum*, Clec: *Cimex lectularius*, Dmel: *Drosophila melanogaster*, Hsap: *Homo sapiens*, Nlug: *Nilaparvata lugens*. (**B**) Evaluation of expression of AmKir channel isotypes in honeybee tissues and life stage. Tissue-specific expression of the AmKir channel isotype was determined using RT-PCR. All honeybee tissue samples underwent identical preparation steps, from dissection to gel electrophoresis. The sizes of the ladder marker next to the fist blot is the same for all the other blots, and are indicated in base pairs (bp). Abbreviations: LE: legs, LA: larvae, HE: heads, BR: brains, GAG: ganglia, GU: guts, MU: muscles, ANT: antennae (**C**) Sequence alignments of the three current-generating AmKir channel isoforms were performed using MegAlign from Lasergen. The blue squares indicate the hydropathy estimation of the transmembrane regions. The red square represents the estimated region of the selectivity filter. The black squares indicate arginine-glutamate salt bridge interactions forming the PIP_2_-binding site in the cytoplasmic domain.

Figure 1A shows the phylogenic tree of Kir genes in humans (black) and insects, including *Apis mellifera*, *Drosophila melanogaster*, and *Aedes aegypti*. For insects, three Kir genes are shown; Kir1 (blue), Kir2 (purple), and Kir3 (orange). The data depicted in Fig. 1B from the RT-PCR analysis reveal that the expression of RNAs corresponding to the AmKir channels across all the honeybee tissues examined. These tissues include adult legs, heads, brains, ganglia, viscera, muscles dissected in the thoracic region and antennae. Additionally, the presence of AmKir channel RNA was detected in larvae. These findings strongly suggest that the AmKir channel plays a significant role in the physiological functions of honeybees. The high sequence similarity between AmKir2.1 and AmKir2.2 did not allow us to design a specific oligonucleotide to assess the tissular expression of AmKir2.2 (Fig. S1).

The data depicted in Fig. 1C show the protein sequences of the three isoforms that generate functional currents (see below). The only variation observed between the AmKir2.2 and AmKir2.3 isotypes was in the short N-terminal tail. The sequence of the selectivity filter motif (glycine-tyrosine-glycine, GYG) was the same for all three isoforms. AmKir2.2 and AmKir2.3 differed from AmKir1.1 in several respects. AmKir1.1 had a longer N-terminal tail and a shorter C-terminal tail. Some similarities were observed in the long C-terminal tail downstream from the transmembrane region. For the pore region, the characteristic GYG motif was conserved in all three sequences. However, the amino acids surrounding the GYG motif in AmKir1.1 were different from those in AmKir2.2 and AmKir2.3, as shown in Fig. 1C. A sequence comparison was conducted between Human Kir (hKir) 1 and 2 and AmKir1 and 2. In Fig. 1C, black squares represent arginine-glutamate salt bridge interactions, delineating the formation of the PIP2-binding site within the cytoplasmic domain.

### Electrophysiological characterization of the AmKir1.1, AmKir2.2, and AmKir2.3 isoforms

**Figure 2:**
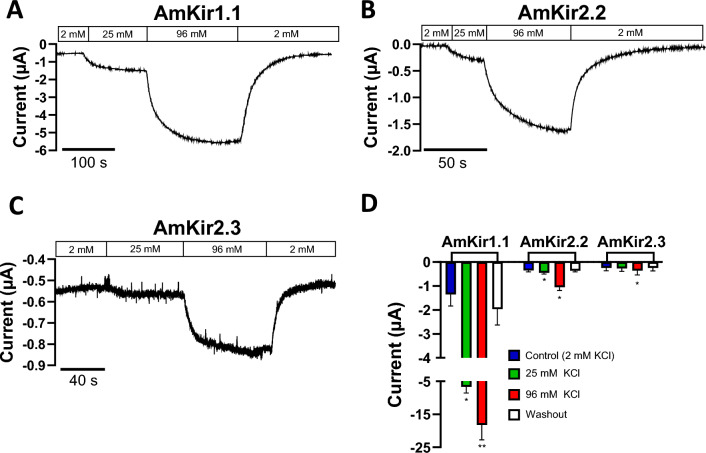
Comparison of the current amplitudes of *Apis mellifera* AmKir channels. (**A**–**C**) Representative raw current traces of the three *Apis mellifera* AmKir channel isoforms at a holding potential of − 80 mV under different conditions: Control (2 mM KCl), 25 mM KCl, 96 mM KCl, and washout (2 mM KCl). (**D**) Comparison bar plots of current amplitudes observed for the three channels tested in (**A**), (**B**), and (**C**) (AmKir1.1 n = 9, AmKir2.2 n = 12, AmKir2.3 n = 6, mean ± SEM). In the AmKir1.1 section, *p < 0.05 and **p < 0.005 vs. AmKir1.1 control. One-way ANOVA multiple comparison test. In the AmKir2.2 section, *p < 0.05 vs. AmKir2.2 control. One-way ANOVA multiple comparison test. In the AmKir2.3 section, *p < 0.05 vs. AmKir2.3 control. One-way ANOVA multiple comparison test.

The data shown in Fig. 2 made it possible to determine the current magnitude from the three isoforms at − 80 mV when they were exposed to different extracellular KCl concentrations (2 mM, 25 mM, and 96 mM). Figure 2A–C are representative raw current traces obtained from AmKir1.1, AmKir2.2, and AmKir2.3, respectively, for the three extracellular KCl concentrations, with a washout with the initial 2 mM KCl control solution. For AmKir1.1, the mean currents for the 2 mM and 25 mM KCl conditions were 7.4% and 36.7%, respectively, of the mean current of the 96 mM KCl condition (18.21 ± 4.53 µA) (Fig. 2D, AmKir1.1 section). For AmKir2.2, the currents in control (R), 25 mM KCl, and 96 mM KCl conditions were smaller than for the equivalent AmKir1.1 conditions. The mean currents for the control and 25 mM KCl conditions were 27.9% and 37.5%, respectively, of the mean current for the 96 mM KCl condition (1.04 ± 0.13 µA) (Fig. 2D, AmKir2.2 section). For AmKir2.3, the currents in the control and 25 mM KCl conditions were 45% and 54.6%, respectively, of the mean current for the 96 mM KCl condition (0.75 ± 0.27 µA) (Fig. 2D, AmKir2.3 section).

**Figure 3:**
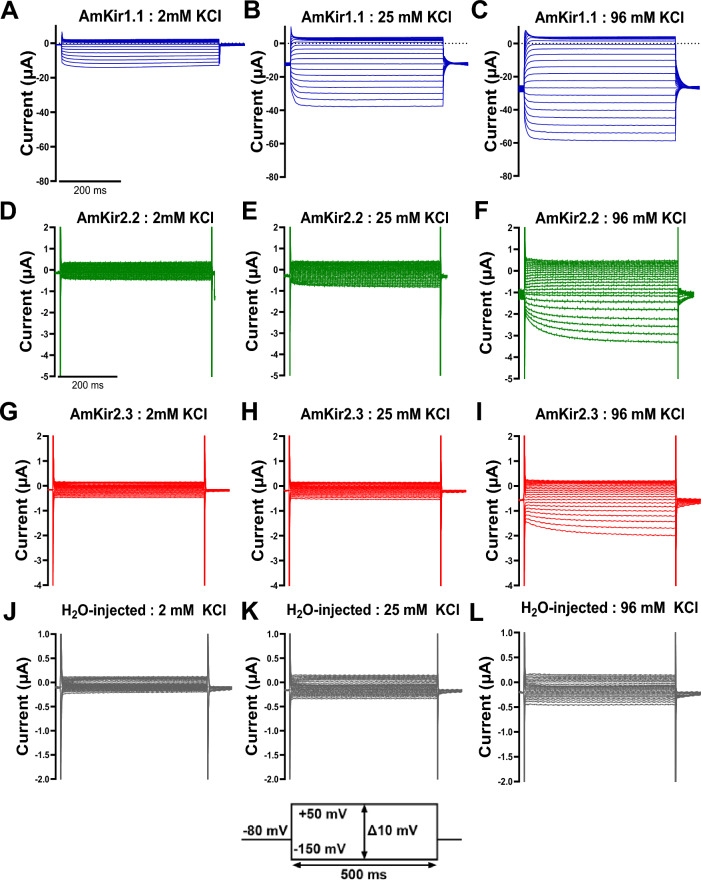
Visual comparison of raw current traces of *Apis mellifera* AmKir channels and H_2_O-injected *Xenopus* oocytes in 2 mM KCl, 25 mM KCl, and 96 mM KCl. (**A**–**C**) Raw current traces of AmKir1.1, (**D**–**F**) AmKir2.2, (**G**–**I**) AmKir2.3 and (**J**–**L**) oocytes H_2_O-injected.

**Figure 4:**
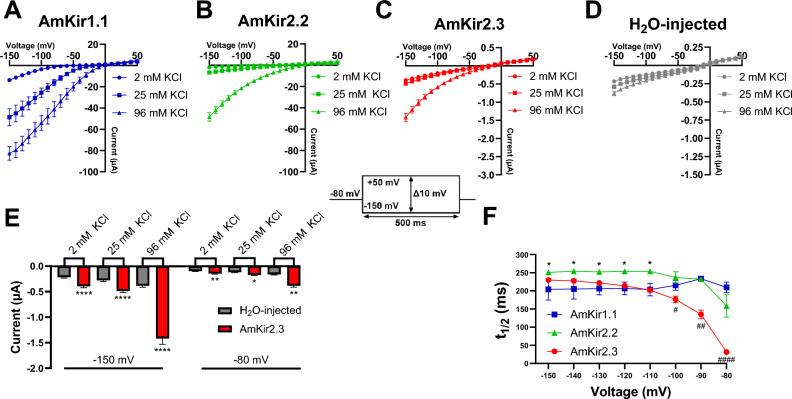
Comparison of I-V curves of *Apis mellifera* AmKir channels and H_2_O-injected *s* oocytes in 2 mM KCl, 25 mM KCl, and 96 mM KCl. (**A**) Curves of I/V curves from AmKir1.1 (n = 9, mean + SEM), (**B**) AmKir2.2 (n = 6, mean + SEM), (**C**) AmKir2.3 (n = 24, mean + SEM) and (**D**) H_2_O-injected *Xenopus* oocytes (n = 19, mean + SEM). (**E**) Current amplitude comparison between AmKir2.3 and H_2_O-injected *Xenopus* oocytes at − 150 mV and − 80 mV for each KCl external concentration condition, *p < 0.05, **p < 0.005, ****p < 0.0001 vs H_2_O-injected. Two-Way ANOVA multiple comparison test (mean + SEM). (**F**) Half-time to peak (t_1/2_) of the three AmKir channels while perfused with 96 mM KCl solution, *p < 0.05 vs. AmKir1.1. Two-way ANOVA multiple comparison test.

**Figure 5:**
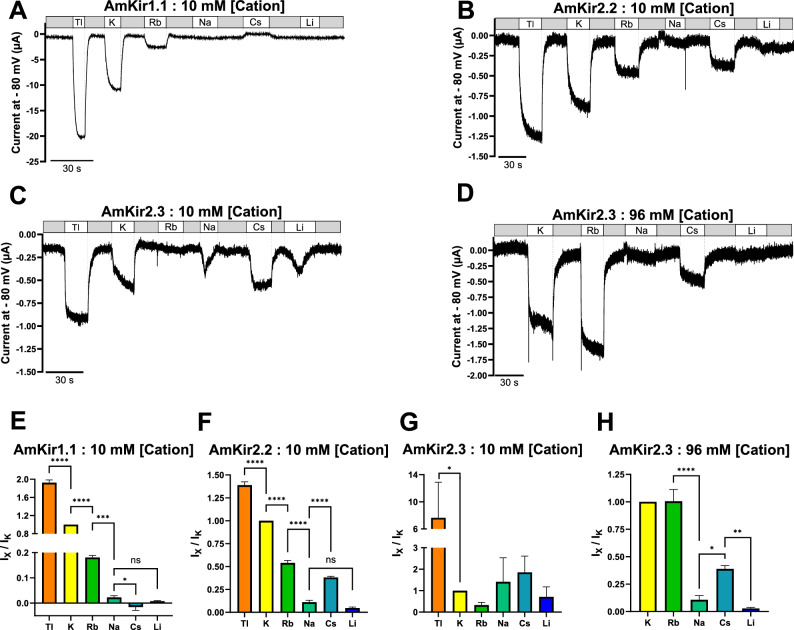
Ionic permeability rankings of the *Apis mellifera* AmKir channel isoforms. (**A**–**D**) Representative raw current traces recorded at a holding potential of − 80 mV with 10 mM of specific monovalent cations including, thallium, potassium, rubidium, sodium, cesium, and lithium (**A**–**C**) and 96 mM of potassium, rubidium, sodium, cesium and lithium (**D**). The grey segment represents the perfusion time of a Ringer’s analog solution without any monovalent cation. (**E**–**H**) Average peak current intensity as a function of the cation used. Data are normalized to the mean potassium peak current. Mean ± SEM, *p < 0.05, **p < 0.005, ***p < 0.0005, ****p < 0.00005. One-way ANOVA multiple comparison test.

The raw current traces (Fig. 3A–L) are representative of the currents generated by AmKir1.1 (Fig. 3A–C), AmKir2.2 (Fig. 3D–F), AmKir2.3 (Fig. 3G–I) and H_2_O-injected oocytes (Fig. 3J-L). The oocytes were subjected to a holding potential of − 80 mV and a stimulation pulse protocol ranging from − 150 mV to + 50 mV with 10-mV increments between each pulse. The orders of magnitude of the currents recorded from the three isoforms differed significantly. AmKir1.1 exhibited an order of magnitude of several tens of µA, while the order of magnitude is a few µA for AmKir2.2, approximately 1 µA for AmKir2.3, and a tenth of a µA for the H_2_O-injected condition. Global inwardly rectifying patterns were observed for the three AmKir channels, with a visible smaller current amplitude observed for AmKir2.3 compared to AmKir2.2. Figure 4A–D shows the average current–voltage (I/V) curves of AmKir1.1, AmKir2.2, AmKir2.3 and H_2_O-injected oocytes. AmKir1.1 exhibited a current with an order of magnitude of several tens of µA in 96 mM KCl (Fig. 4A), while those for AmKir2.2 were of the order of a few µA in 2 and 25 mM KCl, and tens of µA for 96 mM KCl (Fig. 4B) while those for AmKir2.3 were of the order of the µA for the three conditions (Fig. 4C). The order of magnitude for the H_2_O-injected condition was near the tenths of µA for the three conditions Fig. 4D. The current amplitudes between AmKir2.3 and H_2_O-injected are compared across the three external KCl concentrations and at voltage of − 150 V and − 80 mV. AmKir2.3 shows a significantly greater current amplitude than that of H_2_O-injected for each concentration at both tested voltages (Fig. 4E). The I/Vs of AmKir1.1, AmKir2.2 and AmKir2.3 presented an overall inwardly rectifying pattern, while the I/Vs of H_2_O-injected did not presented a rectifying pattern. In line with these types of potassium channels, the opening kinetics were notably swift and displayed minimal voltage dependence. Nevertheless, disparities were observed between AmKir1.1, AmKir2.2 and AmKir2.3. AmKir1.1 exhibited more rapid activating currents from − 150 to − 100 mV. The kinetics of the two channels were identical from − 100 to + 50 mV. AmKir2.3 did not exhibit a significantly different activation kinetics compared to AmKir1.1 and AmKir2.2 from − 150 to − 100 mV but showed a faster kinetic than AmKir2.2 from − 100 to − 80 mV (Fig. 4F). Figure 5 shows the differences in permeability specific to AmKir1.1, AmKir2.2, and AmKir2.3. These permeability tests were performed using the following monovalent cations: thallium, potassium, rubidium, sodium, cesium, and lithium (Fig. 5A–D), and the osmotic balance in the extracellular medium was maintained using NMDG^+^, a non-permeant cation. Grey segments represent perfusion with the Ringer’s control solution (2 mM KCl). Oocytes were maintained at a constant holding potential of − 80 mV. The extracellular monovalent cation concentrations tested were 10 mM for AmKir1.1 and AmKir2.2, and 10 mM and 96 mM for AmKir2.3. The results were normalized to the current amplitude obtained for the potassium condition. The recordings from AmKir1.1 show a basal current in the grey segment that was reduced in the cesium condition. The order of permeability for AmKir1.1 was thallium > potassium > rubidium > sodium > lithium. Cesium slightly inhibited the background potassium current. AmKir1.1 was not permeable to cesium (Fig. 5A). Similarly, the order of permeability for AmKir2.2 was thallium > potassium > rubidium > cesium > sodium = lithium (Fig. 5B), while the order of permeability for AmKir2.3 was thallium > potassium > rubidium > cesium > sodium = lithium (Fig. 5C,D). The recordings for AmKir2.2 and AmKir2.3 showed that both channels are permeable to cesium (Fig. 5B–D). For AmKir1.1, the current amplitude obtained with the thallium condition was 92.2 ± 5.9% greater than with the potassium condition. The rubidium, sodium, and lithium conditions were, respectively, 18 ± 0.7%, 2.2 ± 0.6%, and 0.7 ± 0.2% of the current obtained with the potassium condition (Fig. 5E). For AmKir2.2, the current amplitude obtained with the thallium condition was 26.4 ± 0.03% greater than with the potassium condition. The rubidium, cesium, sodium, and lithium conditions accounted for 53.9 ± 0.02%, 38.05 ± 0.01%, 11.06 ± 0.02%, and 4.68 ± 0.01% of the current obtained with the potassium condition, respectively (Fig. 5F). There were no significant differences between the potassium and rubidium conditions (Fig. 5G,H). The current amplitude of the potassium condition was 13.6 ± 3.4% that of the thallium condition. The current amplitude of the cesium condition was 38.8 ± 3% that of the potassium condition and was significantly different from the sodium and lithium conditions, which exhibited no significant differences (Fig. 5H).

### Characterization of the pharmacology of the AmKir1.1, AmKir2.2, and AmKir2.3 isoforms

**Figure 6:**
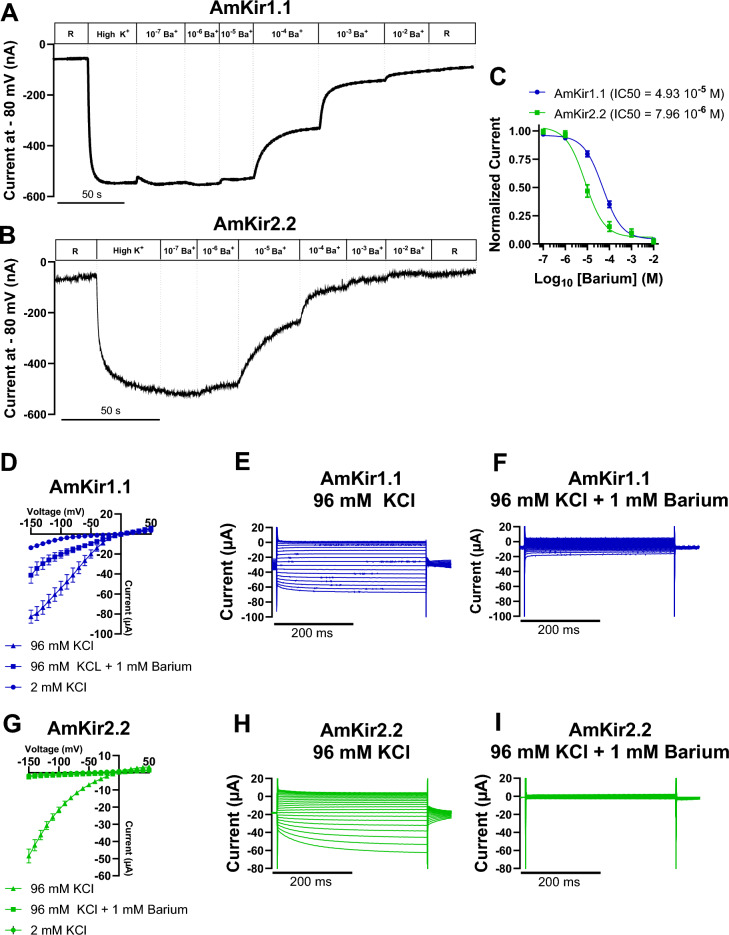
Blockage of *Apis mellifera* AmKir1.1 and AmKir2.2 isoforms by barium. (**A**–**B**) Representative raw current traces of the pharmacological inhibition of AmKir1.1 and AmKir2.2 currents in the presence of increasing concentrations of barium (10^−7^–10^−2^ M). (**C**) Dose-responses curves of the inhibition of AmKir1.1 and AmKir2.2 currents in the presence of increasing barium concentrations (mean ± SEM). (**D**) I/V curves of the AmKir1.1 channel in the presence of high potassium (96 mM KCl), high potassium + barium (96 mM KCl + 1 mM barium), and washout (2 mM KCl) conditions (mean ± SEM). (**E**, **F**) Current traces of the AmKir1.1 channel in the presence of high potassium (96 mM KCl) and high potassium + barium (96 mM KCl + 1 mM barium) conditions, respectively. (**G**) I/V curves of the AmKir2.2 channel in the presence of high potassium (96 mM KCl), high potassium + barium (96 mM KCl + 1 mM barium), and washout (2 mM KCl) conditions (mean ± SEM). (**H**, **I**) Current traces of the AmKir2.2 channel in the presence of high potassium (96 mM KCl) and high potassium + barium (96 mM KCl + 1 mM barium) conditions, respectively.

**Figure 7:**
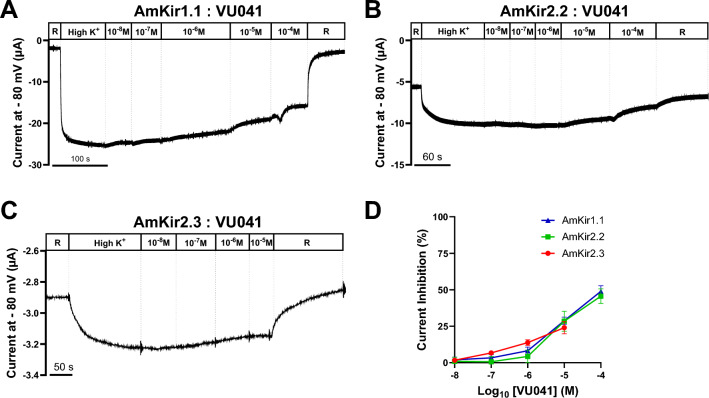
Blockade of *Apis mellifera* AmKir channel isoforms by the insecticide VU041. (**A**–**C**) Representative raw current traces of the pharmacological inhibition of AmKir1.1, AmKir2.2, and AmKir2.3 currents by increasing concentrations of VU041 (10^−8^–10^−4^ M for AmKir1.1 and AmKir2.2 and 10^−8^–10^−5^ M for AmKir2.3). (**D**) Dose–response curves of the inhibition of AmKir1.1, AmKir2.2, and AmKir2.3 currents in the presence of increasing concentrations of VU041 (mean ± SEM, AmKir1.1: n = 12, AmKir2.2: n = 7, AmKir2.3: n = 5).

**Figure 8:**
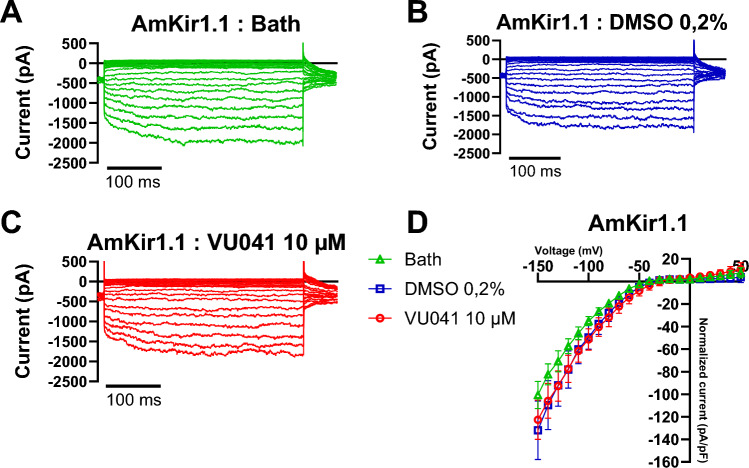
Blockade of *Apis mellifera* AmKir channel isoforms expressed in HEK293T by the insecticide VU041. (**A**–**C**) Comparison of current potential traces of *Apis mellifera* AmKir1.1 in the presence of the bath, 0.2% DMSO, and 10 µM VU041 conditions, respectively. (**D**) I/V curves of the AmKir1.1 channel isoform in the presence of the bath, 0.2% DMSO, and 10 µM VU041 conditions (mean + SEM, Bath n = 9, 0.2% DMSO n = 5, 10 µM VU041).

Figure 6 shows the inhibition of the potassium currents of AmKir1.1 and AmKir2.2 by barium. The effect of barium was studied using raw traces, dose–response curves (Fig. 6A–C), and I/V curves (Fig. 6D–I). The raw traces (Fig. 6A,B) are representative of the current traces recorded with AmKir1.1 and AmKir2.2, with increasing barium concentrations in 96 mM KCl. Stable currents for each barium concentration were used to plot the dose–response curves and determine the pharmacological constants. The dose–response curves indicated that there is a significant difference in the 50% inhibitory dose (IC_50_), with values of 4.93 10^−5^ M and 7.96 10^−6^ M for AmKir1.1 and AmKir2.2, respectively (Fig. 6C). For AmKir1.1, the average current amplitude of the I/V curves at a voltage of − 80 mV for the 2 mM KCl condition was 2.95 ± 1.12 µA whereas, for the 96 mM KCl and 96 mM KCl + 1 mM barium conditions, the current amplitudes were 41.10 ± 7.10 µA and 15.76 ± 2.77 µA, respectively (Fig. 6D–F). For AmKir2.2, the average current amplitude of the I/V curves at − 80 mV for the 2 mM KCl condition was 0.30 ± 0.05 µA, while the current amplitudes were 14.55 ± 1.10 µA and 1.00 ± 0.08 µA, respectively, for the 96 mM KCl and 96 mM KCl + 1 mM barium conditions (Fig. 6G–I). Figure 7 shows the inhibition of the potassium currents of AmKir1.1, AmKir2.2, and AmKir2.3 by VU041. The effect of VU041 was studied using raw traces (Fig. 7A–C) and concentration-percentage of inhibition curves (Fig. 7D). The raw traces are representative of those used to plot the concentration-percentage of inhibition curves. No differences were observed for the three channels for VU041. Curves are analyzed for AmKir1.1, AmKir2.2, and AmKir2.3. Percentage of inhibition were determined and were 28.85 ± 5.46%, 28.46 ± 6.84% and 24.04 ± 1.92% for AmKir1.1, AmKir2.2, and AmKir2.3, respectively, at a VU041 concentration of 10^−5^ M, and 48.94 ± 3.79% and 45.65 ± 5.01% for AmKir1.1 and AmKir2.2, respectively, at a VU041 concentration of 10^−4^ M. No statistical differences were observed between the inhibition percentage of the three isotypes for all VU041 condition. Figure 8 depicts the results regarding the inhibition of potassium currents of AmKir1.1 expressed in HEK293T cells by VU041 (see Fig. 8A–C). The impact of VU041 was assessed by examining current traces under three conditions: Bath, DMSO 0.2% and VU041 10 µM. Figure 8D illustrates the comparison of mean current amplitudes across these three conditions, revealing no significant differences.

## Discussion

The actual ecological conditions present a considerable challenge to the survival of arthropods, especially for vital pollinators like honeybees. The regulation of potassium homeostasis is crucial for arthropod survival and represents a major objective for various organs. Both salivary glands and Malpighian tubules, an organ serving as excretory renal tissue, play a fundamental role in the regulation of this homeostasis. At the molecular level, this regulation is facilitated by inward rectifying potassium channels (Kir channels). Exposure of K_ATP_ channels, a subtype of Kir channels that plays a fundamental role in the viral protection of honeybees^15^, to pharmacological activators induces an increase in immunocompetence and a reduction in mortality associated with viral infections in honeybees^20^. Conversely, several small molecules with insecticidal activity are being developed to target Kir channels and disrupt potassium homeostasis. The functioning of Kir channels in arthropods such as the mosquito *Aedes aegypti*^6^, the aphid *Aphis gossypii*^7^, and the fly *Drosophila melanogaster*^8^ has been the subject of many reports. The Vanderbilt University High Throughput Screening Facility has developed small molecule inhibitors of Kir channels with insecticidal activity such as VU590, VU573 and VU625, which can be used to target the Kir channels of mosquitoes^6,21,22^. Nevertheless, despite the presumed existence of these channels in *Apis mellifera*, no study has yet determined the electrophysiological or pharmacological characteristics of *Apis mellifera* AmKir channels despite the well-documented effect of some small molecules with insecticidal activity on arthropod Kir channels. Only one study has demonstrated that exposure to the small molecule VU041 presents no toxic effects on *Apis mellifera* survival within colonies^16^. Our objective was thus to identify and characterize these channels using electrophysiological and pharmacological methods to ascertain their distinct biophysical properties and evaluate their sensitivity to known blockers such as barium and cesium and potential blockers such as VU041.

Our findings indicate that *Apis mellifera* expresses only two types of Kir channels, namely AmKir1 and AmKir2, which can produce two and four isoforms, respectively. These AmKir isoforms were detected in multiple organs of the multiple honeybees analyzed in our study. Data from Fig. 1B indicates the presence of all isoforms in the brain. From the biological material pool used for RT-PCR, qPCR experiments were conducted to assess quantitative expression of the AmKir channels within each studied organ (Fig. S2). These results imply that inhibiting AmKir channels could lead to specific dysfunctions in bee physiology. Preliminary results indicated that AmKir1.2, AmKir2.1, and AmKir2.4 do not generate functional currents when expressed alone in *Xenopus laevis* oocytes (data not shown). We thus did not perform a functional characterization of these three isoforms. Sequence alignments of these three isoforms revealed that there is a significant similarity between AmKir2.2 and AmKir2.3, both of which share a similar pore region motif. The only striking difference between these two isoforms was the length of the N-terminal tail, with a 31-amino-acid shorter tail for AmKir2.3. The crystal structures of human Kir channels show that the N-terminal and C-terminal tails come together to create a cytoplasmic domain^23^. It has been suggested that this assembly may be present in all Kir channels^24^. As Kir channels are organized as tetramers, the architecture of the four assembled groups of N-terminal and C-terminal tails join the cytoplasmic-side of the pore to extend the ion conduction pathway^25^. These structural differences may contribute to the differences in expression levels observed with the AmKir isoforms. The locations of the transmembrane segments and the pore regions were determined from a Blast-Hydropathy profile analysis using Uniprot software. The pore regions contained the GYG motif, which is conserved in the selectivity filter in Kir channels in general^26^. AmKir2.2 and AmKir2.3 both generated less current than AmKir1.1 (Fig. 2A–C). However, experimentally, AmKir2.3 exhibited a current that was difficult to investigate, most probably because of its very little current density, that we assume related to the low expression level. The clearly different current amplitude that the AmKir2.3 current displays on its I/V curve leaves room for an assumption that variations between AmKir2.2 and AmKir2.3 in the short N-terminal tail might be at the root of a decrease in the expression or stability in the AmKir2.3 cytoplasmic domain structure, which might alter its biophysical properties. This assumption is based on the observations presented in Figs. 3G–I and 4C,F, where we recorded a smaller current amplitude and different opening kinetic than AmKir2.2. A crystal structure analysis of the prokaryotic KirBac1.1 channel^27^ revealed that the N-terminal tail contains a sliding helical segment situated in proximity and parallel to the cytoplasmic side of the membrane. Previous studies have shown that this N-terminal sliding helix interacts with membrane phospholipids and the C-terminal portion of the cytoplasmic domain through β-sheet interactions at the N-terminal end, thereby tightly regulating channel gating^28^. Additionally, the characteristic inwardly rectifying pattern of Kir channels, which is influenced by intracellular magnesium or polyamines, has been linked to specific residues located in the second transmembrane helix and in the cytoplasmic domain^29^. We thus hypothesized that the differences of current amplitude and opening kinetics of AmKir2.3 are solely attributed to its shorter N-terminal segment compared to AmKir2.2. This discrepancy would result in a lack of interaction between the N-terminal sliding helix and the tail end with the remainder of the cytoplasmic domain. Given the smaller current amplitude exhibited by AmKir2.3 compared to AmKir2.2, we aimed to compare this with current amplitudes from H_2_O-injected oocytes. The objective was to ascertain whether the channel was adequately expressed in our system and if the recorded current traces indeed originated from the overexpressed channel rather than the oocyte's endogenous currents. Our findings indicate that irrespective of the voltage or external KCl concentration, the current derived from AmKir2.3 was significantly greater than that from H_2_O-injected oocytes and exhibited inward rectifying properties (Fig. 4E). This disparity confirms that our experiments on AmKir2.3 reliably capture currents carried from AmKir2.3 and not from the oocyte's endogenous currents. The channel kinetics observed (Fig. 4F) were consistent with the kinetics reported in the literature. Although they were not specifically analyzed, current traces suggested that, in other arthropods such as *Drosophilia melanogaster*^8^, dKir1 and dKir2 have different activation kinetics, with dKir2, like AmKir2, exhibiting slow activation kinetics. However, it is well-established that phosphatidylinositol 4,5-bisphosphate (PIP2) acts as an activator of Kir channels^30,31^. PIP2 interacts with Kir channels through a PIP2-binding site located at the cytoplasmic domain interface^32^. This interaction is facilitated by a salt bridge interaction between arginine and glutamate residues. The disruption or modification of these salt bridges affects the sensitivity of PIP2 for its binding site, influencing the probability of the channel opening^33^. We conducted a sequence comparison of the salt bridge regions between hKir1.1 and AmKir1.1 and between hKir2.2 and AmKir2.2, which revealed differences in both length and sequence (Fig. 1C, black rectangle). We hypothesized that these variations in length and sequence between AmKir1.1 and AmKir2.2 may result in modified PIP2 sensitivity, potentially explaining the differences in kinetics observed between these two isoforms. In addition, the characterizations of the properties of the three AmKir channels indicated that the results of the permeability experiments are consistent with the fact that the pore amino acid sequences of AmKir2.2 and AmKir2.3 are identical but are different from those of AmKir1.1. AmKir1.1, unlike AmKir2.2 and AmKir2.3, is not permeable to cesium (Fig. 5A,E). However, it was clear that AmKir2.2 and AmKir2.3 are permeable to cesium (Fig. 5F,H), which has not often been reported in the literature. However, our results align with those obtained with mosquitoes, where AeKir1 did not show cesium permeability, whereas AeKir2 did^34^. Overall, our results align with expectations for a Kir channel, demonstrating high permeability to potassium and thallium, low permeabilities for lithium and sodium, and a relative permeability to cesium for certain isoforms. This could be attributed to variations in the N-terminal and C-terminal sequences between AmKir1.1, AmKir2.2, and AmKir2.3, resulting in differences in their respective cytoplasmic domains. These differences may lead to varying ionic permeabilities, even though the GYG filter motif is conserved. All three isoforms share a common trait in that they all allow the passage of thallium and rubidium but little or no sodium or lithium. Of note, we decided to raise the external concentration of ion to 96 mM for AmKir2.3 experiments (Fig. 5D) because 10 mM traces were too difficult to investigate. However, we could not investigate the thallium current at 96 mM due to a too little solubility factor of thallium-chloride in water (3.3 g/L). The results regarding the pharmacology analyses of the effect of barium on these channels were consistent with those reported in the literature^34^. Due to the low expression of AmKir2.3 we did not investigate the effect of barium in this channel. AmKir1.1 and AmKir2.2 were both blocked by barium, a known non-selective Kir channel blocker^5^. However, a log(IC_50_) comparison indicated that a ten-fold higher concentration of barium was required to block AmKir1.1 than was required to block AmKir2.2 (Fig. 6C). While in mosquitoes and aphids, AeKir1/ApKir1 appear to be more sensitive to barium than AeKir2, we observe the opposite in bees. It is noteworthy that in the pore region, 7 amino acids upstream of the selectivity filter GYG, aphids have a valine, bees have an isoleucine, and mosquitoes have a leucine. Since the blocking action of barium occurs by pore blocking, we hypothesize that this difference in amino acid among these three insects causes structural and affinity modification, and thus the observed differences in barium sensitivity^34,35^. Surprisingly, the effects of VU041 on the AmKir channels of *Apis mellifera* were different from those reported in the literature for the Kir channels of *Aedes aegypti* and *Aphis gossypii*. Although a VU041concentration of 10^−6^ M was sufficient to completely block the channel activity^16^ of *Aedes aegypti*, a concentration of 10^−5^ M, on average, do not even cause a 50% inhibition for the three AmKir isoforms (Fig. 7D). We could not experimentally use concentrations above 10^−4^ M due to a potential effect of DMSO, the solvent in which VU041 is dissolved. We also tested VU041 on AmKir1.1 channels expressed in HEK293 cells but observed no significant effect up to 10^−5^ M (Fig. 8). We know that the Kir1 ortholog of the *Aedes aegypti* shares only 55% of amino acid identity with the Kir1 of *Apis mellifera*^36^. The VU041 interaction site with the AeKir channel remains unknown, it may be located on a non-conserved region of the channels of *Apis mellifera* and *Aedes aegypti*^16^. Testing VU041 in native cells could provide proof that it does not affect *Apis mellifera* Kir channels. In addition co-expressing multiple monomers at the same time can lead to the heterotetramerization of the channel sub-units, resulting in variations in the biophysical and pharmacological properties of the channel^37^. Obtaining a better understanding of the properties of AmKir homotetramers could be a first step toward studying more complex interactions of AmKir channel monomers. As three of the six AmKir channels (AmKir1.2, AmKir2.1, and AmKir2.4) did not exhibit potassium currents when expressed as homotetramers and as precise heterotetrametric Kir structures are responsible for some physiological pathways^38^, further research on this aspect will be required in the future.

## Conclusion

The present study entailed the cloning, functional expression, and pharmacological characterization of inwardly rectifying potassium channels in *Apis mellifera*. Electrophysiological characterizations were conducted to assess the currents carried by AmKir channels, their respective permeabilities, and their pharmacological roles in characterizing the electrophysiological landscape that surrounds the vital systems of arthropods. The concern with respect to the effects of insecticides on the survival of pollinating arthropods is increasing each year. Knowing that one of these small molecules with insecticidal activity, VU041, is lethal to certain “harmful” arthropods such mosquitoes and aphids but is potentially safe for honeybees opens the door to broader research possibilities in the fields of agriculture, food production, and health pharmacology. This approach underscores the significance of understanding and advancing the physiological, electrophysiological, and pharmacological knowledge base of arthropods to safeguard the survival of pollinating insects.

### Supplementary Information

Supplementary Information 1.

Supplementary Figure S1.

Supplementary Figure S2.

## Data Availability

The data underpinning the findings of this study are accessible from the corresponding author upon reasonable request. Please contact Dr. Mohamed Chahine at Mohamed.chahine@phc.ulaval.ca to inquire about accessing the data.
