# Supplementary material for: Cloning, functional expression, and pharmacological characterization of inwardly rectifying potassium channels (Kir) from Apis mellifera
Source: Sci Rep. 2024 Apr 3;14:7834. doi: 10.1038/s41598-024-58234-0 (PMC10991380; doi:10.1038/s41598-024-58234-0)
Supplement: Supplementary file 1 — Supplementary Information 1. [file 41598_2024_58234_MOESM1_ESM.docx]

**Supplementary Material**

**Methods**

**qPCR and tissue expression**: For qPCR, total RNA from different tissues (*Apis Mellifera*; Larvae, Legs, Head, Brain, Ganglia, Guts, Muscle, and Antennae) was obtained using the Trizol reagent protocol (Life Technologies, Trizol). The total RNA from different tissues was treated with RNase-free DNAseI (New England Biolabs). The cDNA was synthesized using ProtoScript^®^ II First Strand cDNA Synthesis kits (New England Biolabs). PCR was performed using Roche kits (LighCycler^®^ 480 SYBR Green I Master). The oligonucleotides used for qPCR are listed in Table 1. Ct values were obtained in triplicate concerning two reference genes, GAPDH and RSP18, and extractions derived from isolated organs. The ΔCt values relative to the organs of interest and reference genes were juxtaposed against the qPCR efficiency values, subsequently normalized to the values obtained for the muscle tissue. No specific oligonucleotide for AmKir2.2 could be developed to assess quantitative expression of this isoform due to the high sequence similarity between AmKir2.1 and AmKir2.2 (**Figure S2**).

**Table S1**: Oligonucleotide list used for qPCR experiments

| **Gene** | **Primers (5'-3')** | | **Tm (°C)** | **Length** |
| --- | --- | --- | --- | --- |
|  | **Forward** | **Reverse** |  | **(pb)** |
| *AmKir1.1* | TCAGAGACAGCAACAGAGCAG | GTTGGTCAGGATTCCCCGTT | 58 | 149 |
| *AmKir1.2* | AAAGATCCACTTGGGCGAGG | TCGTCTGCCGGTATTTTTGC | 63 | 137 |
| *AmKir2.1* | AGCTGGAAAACGCGGGAAAA | CACCATTCCTCGTCCCCGAA | 58 | 124 |
| *AmKir2.3* | CAACCCGCGCCTGTTATAGT | CGGTACCGACTCCTGCTTC | 63 | 71 |
| *AmKir2.4* | GCGGAGAAATCCATCGAGTC | GAAGCTCAGGCTGAAGCAGA | 63 | 196 |
| *GAPDH* | CACCTTCTGCAAAATTATGGCG | ACCTTTGCCAAGTCTAACTGTTAA | 58 | 188 |
| *RPS18* | GATTCCCGATTGGTTTTTGAATAG | AACCCCAATAATGACGCAAACC | 58 | 152 |

Tm: melting temperature.

**Figure legends:**

**Figure S1:** Sequence alignment of the four AmKir2 channel isoforms. Blue: identical sequences shared by all channels. Green and yellow: specific sequences of AmKir2.3 and AmKir2.4, respectively.

**Figure S2:** Quantitative representation of fold change values of qPCR of AmKir channels in regards of two reference genes, GAPGH and RPS18, and normalized to muscle in different organs and developmental stages of *Apis mellifera*. Abbreviations: LE: legs, LA: larvae, HE: heads, BR: brains, GAG: ganglia, GU: guts, MU: muscles, ANT: antennae. Data were normalized to MU.
