# Supplementary material for: Cloning, functional expression, and pharmacological characterization of inwardly rectifying potassium channels (Kir) from Apis mellifera
Source: Sci Rep. 2024 Apr 3;14:7834. doi: 10.1038/s41598-024-58234-0 (PMC10991380; doi:10.1038/s41598-024-58234-0)
Supplement: Supplementary file 2 — Supplementary Figure S1. [file 41598_2024_58234_MOESM2_ESM.pdf]

```
AmKir 2.1    1  MASVGTISCNGRGNLKQLRVRRAYVRCSSWKTRENGTSLDHGDDPCTYIYIYTPWISVARCYLSGTRNGGIPGTEGRRCGCRRKVRGNEETMAENRTLLSNGRESSSRGRLHLRLHSSWAKKSHRRSSGMEEPQKLFRLP  140
AmKir 2.2       ---------------------------------------------------------------------------------------------------------------------------------MEEPQKLFRLP   11
AmKir 2.3       --------------------------------------------------------------------------------------------------------------------------------------------
AmKir 2.4       --------------------------------------------------------------------------------------------------------------------------------------------

AmKir 2.1  141  GTIEEEEEKDNEDRRRQQLQENPSTADSPLLEQRNTGTLSSLPRYVTLVRVSTQSSSIRSAYRRQDSTRSRYRAWPTRKLRRRAVLKNGDCNVLQSRISRRSLRFLRDIFTTLVDTQWRWMLLCFSLSFVLSWLGFAVIW  280
AmKir 2.2   12  GTIEEEEEKDNEDRRRQQLQENPSTADSPLLEQRNTGTLSSLPRYVTLVRVSTQSSSIGGGVGRQDSTRSRYRAWPTRKLRRRAVLKNGDCNVLQSRISRRSLRFLRDIFTTLVDTQWRWMLLCFSLSFVLSWLGFAVIW  151
AmKir 2.3    1  -------------------MNGNVAKDPISKSSSPDREEKEKLLTSLTQPAPVIVKLQKSTNSSFRSRSRYRAWPTRKLRRRAVLKNGDCNVLQSRISRRSLRFLRDIFTTLVDTQWRWMLLCFSLSFVLSWLGFAVIW  121
AmKir 2.4    1  --------------------------------------------------------------MRRNPSSRYRAWPTRKLRRRAVLKNGDCNVLQSRISRRSLRFLRDIFTTLVDTQWRWMLLCFSLSFVLSWLGFAVIW   78

AmKir 2.1  281  WLIAFSHGDFEERHLPPYQIENNWTPCVNNIFSFTSCFLFSIETQHTIGYGSRGTTEECPEAIFVMCIQSIVGVMIQAFMVGIVFAKMSRPKQRTQTLLFSRNAVICQRDGELCLMFRVGDMRKSHIIGAAIRAQLIRSR  420
AmKir 2.2  152  WLIAFSHGDFEERHLPPYQIENNWTPCVNNIFSFTSCFLFSIETQHTIGYGSRGTTEECPEAIFVMCIQSIVGVMIQAFMVGIVFAKMSRPKQRTQTLLFSRNAVICQRDGELCLMFRVGDMRKSHIIGAAIRAQLIRSR  291
AmKir 2.3  122  WLIAFSHGDFEERHLPPYQIENNWTPCVNNIFSFTSCFLFSIETQHTIGYGSRGTTEECPEAIFVMCIQSIVGVMIQAFMVGIVFAKMSRPKQRTQTLLFSRNAVICQRDGELCLMFRVGDMRKSHIIGAAIRAQLIRSR  261
AmKir 2.4   79  WLIAFSHGDFEERHLPPYQIENNWTPCVNNIFSFTSCFLFSIETQHTIGYGSRGTTEECPEAIFVMCIQSIVGVMIQAFMVGIVFAKMSRPKQRTQTLLFSRNAVICQRDGELCLMFRVGDMRKSHIIGAAIRAQLIRSR  218

AmKir 2.1  421  TTKEGEVLSQNQQELAVGTDGQNGNLFFIWPTTIVHRINEESPFYNMSAEDMLTERFEIVAILEGTIESTGQTTQARSSYLPQEILWGYRFEPMVTYSKERQGYEVDYSLFNSTTQVGTPLCSGRELAEFYKAQEELRHG  560
AmKir 2.2  292  TTKEGEVLSQNQQELAVGTDGQNGNLFFIWPTTIVHRINEESPFYNMSAEDMLTERFEIVAILEGTIESTGQTTQARSSYLPQEILWGYRFEPMVTYSKERQGYEVDYSLFNSTTQVGTPLCSGRELAEFYKAQEELRHG  431
AmKir 2.3  262  TTKEGEVLSQNQQELAVGTDGQNGNLFFIWPTTIVHRINEESPFYNMSAEDMLTERFEIVAILEGTIESTGQTTQARSSYLPQEILWGYRFEPMVTYSKERQGYEVDYSLFNSTTQVGTPLCSGRELAEFYKAQEELRHG  401
AmKir 2.4  219  TTKEGEVLSQNQQELAVGTDGQNGNLFFIWPTTIVHRINEESPFYNMSAEDMLTERFEIVAILEGTIESTGQTTQARSSYLPQEILWGYRFEPMVTYSKERQGYEVDYSLFNSTTQVGTPLCSGRELAEFYKAQEELRHG  358

AmKir 2.1  561  NGTVIVDEDFLTESCQESQCHCGHRATSHHHHHHHHHLNHHPSHHLNYLDSGRSETSADEATTSCRNAYRDSVYHGPPVAIPHRNDNPHFKILDLDPDGIQVMGEIDLQHLPDAVNKEILKNSREIIFEEPETSREGSPL  700
AmKir 2.2  432  NGTVIVDEDFLTESCQESQCHCGHRATSHHHHHHHHHLNHHPSHHLNYLDSGRSETSADEATTSCRNAYRDSVYHGPPVAIPHRNDNPHFKILDLDPDGIQVMGEIDLQHLPDAVNKEILKNSREIIFEEPETSREGSPL  571
AmKir 2.3  402  NGTVIVDEDFLTESCQESQCHCGHRATSHHHHHHHHHLNHHPSHHLNYLDSGRSETSADEATTSCRNAYRDSVYHGPPVAIPHRNDNPHFKILDLDPDGIQVMGEIDLQHLPDAVNKEILKNSREIIFEEPETSREGSPL  541
AmKir 2.4  359  NGTVIVDEDFLTESCQESQCHCGHRATSHHHHHHHHHLNHHPSHHLNYLDSGRSETSADEATTSCRNAYRDSVYHGPPVAIPHRNDNPHFKILDLDPDGIQVMGEIDLQHLPDAVNKEILKNSREIIFEEPETSREGSPL  498

AmKir 2.1  701  LLFKSSANRRSSKHPLLDEERRSLNSSKRSLCHRNILRGCFEEEKRSLDGSRRNLHGSRKYLLIPIDASKVADTGTRENFAKRQTGESRECLSNGGRRMMKGQSKEIAAEPETRRRLNSNDDRLMIEMEKSSKCPARHET  840
AmKir 2.2  572  LLFKSSANRRSSKHPLLDEERRSLNSSKRSLCHRNILRGCFEEEKRSLDGSRRNLHGSRKYLLIPIDASKVADTGTRENFAKRQTGESRECLSNGGRRMMKGQSKEIAAEPETRRRLNSNDDRLMIEMEKSSKCPARHET  711
AmKir 2.3  542  LLFKSSANRRSSKHPLLDEERRSLNSSKRSLCHRNILRGCFEEEKRSLDGSRRNLHGSRKYLLIPIDASKVADTGTRENFAKRQTGESRECLSNGGRRMMKGQSKEIAAEPETRRRLNSNDDRLMIEMEKSSKCPARHET  681
AmKir 2.4  499  LLFKSSANRRSSKHPLLDEERRSLNSSKRSLCHRNILRGCFEEEKRSLDGSRRNLHGSRKYLLIPIDASKVADTGTRENFAKRQTGESRECLSNGGRRMMKGQSKEIAAEPETRRRLNSNDDRLMIEMEKSSKCPARHET  638

AmKir 2.1  841  MLNDEGCGTSLSYPNLSPRSKDGQGSPVPPASNASPESGFYEASAQWNSSPEYGRKQLGLAPPPPNGSPVVARNRRSYENAQLQDVNEALGVSRPNSDNSDSLDSEASSKNPANDGDAPPRRYGEKKQPISYTSV*  975
AmKir 2.2  712  MLNDEGCGTSLSYPNLSPRSKDGQGSPVPPASNASPESGFYEASAQWNSSPEYGRKQLGLAPPPPNGSPVVARNRRSYENAQLQDVNEALGVSRPNSDNSDSLDSEASSKNPANDGDAPPRRYGEKKQPISYTSV*  846
AmKir 2.3  682  MLNDEGCGTSLSYPNLSPRSKDGQGSPVPPASNASPESGFYEASAQWNSSPEYGRKQLGLAPPPPNGSPVVARNRRSYENAQLQDVNEALGVSRPNSDNSDSLDSEASSKNPANDGDAPPRRYGEKKQPISYTSV*  816
AmKir 2.4  639  MLNDEGCGTSLSYPNLSPRSKDGQGSPVPPASNASPESGFYEASAQWNSSPEYGRKQLGLAPPPPNGSPVVARNRRSYENAQLQDVNEALGVSRPNSDNSDSLDSEASSKNPANDGDAPPRRYGEKKQPISYTSV*  773
```
