# Supplementary figures and images for: Cloning, functional expression, and pharmacological characterization of inwardly rectifying potassium channels (Kir) from Apis mellifera
Source: Sci Rep. 2024 Apr 3;14:7834. doi: 10.1038/s41598-024-58234-0 (PMC10991380; doi:10.1038/s41598-024-58234-0)

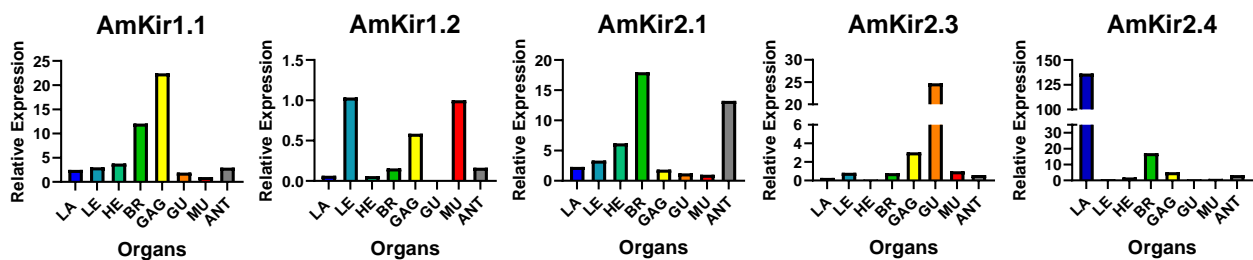

Supplement: Supplementary file 3 — Supplementary Figure S2. [file 41598_2024_58234_MOESM3_ESM.pdf]
